# Minnesota peat viromes reveal terrestrial and aquatic niche partitioning for local and global viral populations

**DOI:** 10.1186/s40168-021-01156-0

**Published:** 2021-11-26

**Authors:** Anneliek M. ter Horst, Christian Santos-Medellín, Jackson W. Sorensen, Laura A. Zinke, Rachel M. Wilson, Eric R. Johnston, Gareth Trubl, Jennifer Pett-Ridge, Steven J. Blazewicz, Paul J. Hanson, Jeffrey P. Chanton, Christopher W. Schadt, Joel E. Kostka, Joanne B. Emerson

**Affiliations:** 1 Department of Plant Pathology, University of California, Davis, Davis, CA USA; 2 Department of Earth, Ocean, and Atmospheric Science, Florida State University, Tallahassee, FL USA; 3 Biosciences Division, Oak Ridge National Laboratory, Oak Ridge, TN USA; 4 Physical and Life Sciences Directorate, Lawrence Livermore National Laboratory, Livermore, CA USA; 5 Environmental Sciences Division, Oak Ridge National Laboratory, Oak Ridge, TN USA; 6 Schools of Biology and Earth & Atmospheric Sciences, Georgia Institute of Technology, Atlanta, GA USA; 7 Center for Microbial Dynamics and Infection, Georgia Institute of Technology, Atlanta, GA 30332 USA; 8 Genome Center, University of California, Davis, Davis, CA USA

**Keywords:** Viral ecology, Viromics, Soil viruses, Soil microbial ecology, Peat, Metagenomics, Biogeography, Virome

## Abstract

**Background:**

Peatlands are expected to experience sustained yet fluctuating higher temperatures due to climate change, leading to increased microbial activity and greenhouse gas emissions. Despite mounting evidence for viral contributions to these processes in peatlands underlain with permafrost, little is known about viruses in other peatlands. More generally, soil viral biogeography and its potential drivers are poorly understood at both local and global scales. Here, 87 metagenomes and five viral size-fraction metagenomes (viromes) from a boreal peatland in northern Minnesota (the SPRUCE whole-ecosystem warming experiment and surrounding bog) were analyzed for dsDNA viral community ecological patterns, and the recovered viral populations (vOTUs) were compared with our curated PIGEON database of 266,125 vOTUs from diverse ecosystems.

**Results:**

Within the SPRUCE experiment, viral community composition was significantly correlated with peat depth, water content, and carbon chemistry, including CH_4_ and CO_2_ concentrations, but not with temperature during the first 2 years of warming treatments. Peat vOTUs with aquatic-like signatures (shared predicted protein content with marine and/or freshwater vOTUs) were significantly enriched in more waterlogged surface peat depths. Predicted host ranges for SPRUCE vOTUs were relatively narrow, generally within a single bacterial genus. Of the 4326 SPRUCE vOTUs, 164 were previously detected in other soils, mostly peatlands. None of the previously identified 202,371 marine and freshwater vOTUs in our PIGEON database were detected in SPRUCE peat, but 0.4% of 80,714 viral clusters (VCs, grouped by predicted protein content) were shared between soil and aquatic environments. On a per-sample basis, vOTU recovery was 32 times higher from viromes compared with total metagenomes.

**Conclusions:**

Results suggest strong viral “species” boundaries between terrestrial and aquatic ecosystems and to some extent between peat and other soils, with differences less pronounced at higher taxonomic levels. The significant enrichment of aquatic-like vOTUs in more waterlogged peat suggests that viruses may also exhibit niche partitioning on more local scales. These patterns are presumably driven in part by host ecology, consistent with the predicted narrow host ranges. Although more samples and increased sequencing depth improved vOTU recovery from total metagenomes, the substantially higher per-sample vOTU recovery after viral particle enrichment highlights the utility of soil viromics.

**Video abstract** The importance of Minnesota peat viromes in revealing terrestrial and aquatic niche partitioning for viral populations

**Supplementary Information:**

The online version contains supplementary material available at 10.1186/s40168-021-01156-0.

## Background

Peatlands store approximately one third of the world’s soil carbon (C) and have a significant role in the global C cycle [1]. Microbial activity in peatlands plays a key role in soil C and nutrient cycling, including soil organic C mineralization to the greenhouse gases, methane (CH_4_), and carbon dioxide (CO_2_) [2–5]. Given the abundance of viruses in soil (10^7^ to 10^10^ per gram of soil [6–9]) and evidence for viral impacts on microbial ecology and biogeochemistry in other ecosystems [10–12], it is likely that viral infection of soil microorganisms influences the biogeochemical and C cycling processes of their hosts [13–15]. In marine ecosystems, viruses are estimated to lyse 20–40% of ocean microbial cells daily, impacting global ocean food webs and the marine C cycle [16–18], and viral contributions to terrestrial ecosystems are presumed to be similarly important but are less well understood [6, 13, 14, 19–21].

Our current understanding of soil viral ecology stems from pioneering studies on viral abundance, morphology, amplicon sequencing, and lysogeny of bacteria [22–27], along with early viral size-fraction metagenomic (viromic) investigations [28–30]. More recently, total soil and wetland metagenomic datasets have been mined for viral sequences [10, 15, 31], revealing thousands of previously unknown viral populations (vOTUs) and suggesting habitat specificity for some of these viruses. Metatranscriptomic data mining has recently been used to explore RNA viral communities, revealing differences in bulk, rhizosphere, and detritusphere (plant litter-influenced) soil compartments [32], along with potential viral contributions to the ecology of the *Sphagnum* moss microbiome [33]. In addition to mining omic data for viral signatures, viromics (the laboratory enrichment of viral particles prior to DNA extraction and metagenomic sequencing) has recently been paired with high-throughput sequencing to investigate viral communities in soil [13, 15, 34, 35]. Although we now have an array of laboratory and bioinformatics methods for soil viral ecology [7, 15, 23, 31, 34, 36–41], we lack a thorough comparative understanding of these approaches and best practices.

Thawing permafrost peatlands have been the focus of several recent studies of viral diversity and virus–host dynamics, in order to better understand the ecological patterns underlying C emissions from these climate-vulnerable ecosystems [13, 15, 42–44]. Thawing permafrost peat has been characterized by relatively high viral diversity (thousands of vOTUs), including viruses predicted to infect methanogens and methanotrophs responsible for CH_4_ cycling [15]. Evidence for more direct viral impacts on ecosystem C cycling has been revealed by the recovery of putative viral auxiliary metabolic genes (AMGs) [13, 15], specifically, virus-encoded glycosyl hydrolases capable of degrading complex C into simple sugars [15]. Although we are gaining insights into soil viral ecology within specific ecosystems, our understanding of global soil viral biogeographical patterns is limited and is thus far derived predominantly from cultivation-based efforts [44, 45].

In this study, we examined peat viral communities at the southern edge of the boreal zone in the Marcell Experimental Forest (MEF) in Minnesota, USA [46, 47]. MEF has been the site of numerous studies on greenhouse gas emissions, C sequestration, hydrology, biogeochemistry, and vegetation [48–53]. To investigate the response of peatlands to increasing temperature and atmospheric CO_2_ concentrations, the US Department of Energy (DOE) established the Spruce and Peatland Responses Under Changing Environments (SPRUCE) experiment in MEF. This experiment is within an intact peat bog ecosystem, consisting of *Picea mariana* (black spruce) and *Larix laricina* (larch) trees, an ericaceous shrub layer, and a predominant cover of *Sphagnum* with minor contributions of other mosses [46, 47, 54]. SPRUCE researchers are studying whole-ecosystem responses to temperature and elevated CO_2_ (eCO_2_), including the responses of plants, above- and belowground microbial communities, and whole-ecosystem processes, such as greenhouse gas emissions [1, 46, 47, 55–59], but as yet, the peat viral communities in this experiment remain unexplored.

Here, we used a combination of total soil metagenomics and viromics to (1) investigate peat viral community composition and its potential drivers in the SPRUCE experiment, (2) place the recovered vOTUs in biogeographical and ecosystem context, and (3) compare the two approaches (total metagenomics and viromics) for recovering soil viral population sequences. We are also contributing a new database for reference-based viral genome recovery: the *P*hages and *I*ntegrated *G*enomes *E*ncapsidated *O*r *N*ot (PIGEON) database of 266,125 vOTU sequences from diverse ecosystems.

## Results and discussion

### Dataset overview and peat viral population (vOTU) recovery

To improve our understanding of peat viral diversity, we leveraged 82 peat metagenomes from cores collected from the SPRUCE experiment in northern Minnesota, USA in 2015 and 2016, along with five paired viromes and metagenomes that we collected along a transect outside the experimental plots from the same bog in 2018 at near-surface (top 10 cm) depths. In the field experiment, deep peat heating (DPH) and whole-ecosystem warming (WEW) treatments heated the peat (to a depth of 2 m) and air inside 8 chambered enclosures (two per treatment) to target temperatures of + 2.25, + 4.5, + 6.75, and + 9 °C above ambient temperature [1, 47, 54, 60]. There were also two ambient experimental chambers and two unchambered ambient plots (Table S1). Peat samples for metagenomics were collected from four depths (10–20 cm, 40–50 cm, 100–125 cm, and 150–175 cm) per year in each chamber and unchambered ambient plot (38 and 44 total soil metagenomes were successfully sequenced in 2015 and 2016, respectively), with approximate sequencing depths of 6 Gbp per metagenome in 2015 and 15 Gbp in 2016. From each of the five transect peat samples (Supplementary Figure 1), a viral size-fraction metagenome (virome) and total soil metagenome were sequenced, each to a depth of approximately 14 Gbp.

Reads from the SPRUCE experiment metagenomes (82), transect viromes (5), and transect total soil metagenomes (5) were assembled into contigs ≥ 10 kbp, from which viral contigs were identified [38, 39] and clustered into 5006 species-level viral populations (viral operational taxonomic units (vOTUs) [61]). These vOTUs were then clustered with 261,799 vOTUs from diverse habitats in our PIGEON database (see methods, Table S2) [10, 13, 15, 31, 34, 62–66]. The resulting clustered database of 266,125 “species-level” vOTUs was used as a reference for read mapping from each of our metagenomes. In total, we detected 4326 vOTUs through read mapping from the SPRUCE experiment and adjacent peatlands, and of these, 17.3% were recovered by both VirSorter and DeepVirFinder, 52.3% were recovered by VirSorter alone, and 30.4% were recovered by DeepVirFinder alone. Henceforth, “SPRUCE” refers to our data from the SPRUCE experiment and/or transect, unless otherwise specified.

### Investigating patterns and potential drivers of peat viral community composition in the SPRUCE experimental plots

**Fig. 1:**
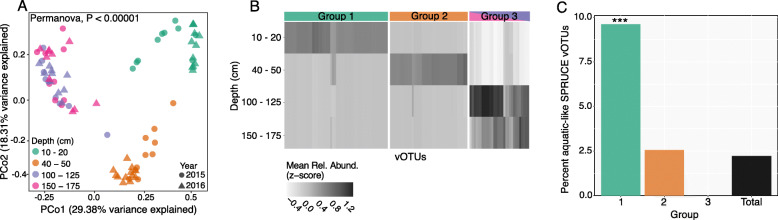
Peat viral community and population (vOTU) abundance patterns with depth in the SPRUCE experimental plots. **A** Principal coordinates analysis (PCoA) of viral community composition in 82 samples (total soil metagenomes) from peat bog soil from the Marcell Experimental Forest in northern Minnesota (USA) collected from the SPRUCE experimental plots and chambers (temperature treatments ranging from ambient to + 9 °C above ambient), based on Bray-Curtis dissimilarities derived from the table of vOTU abundances (read mapping to vOTUs, *n* = 2699). Each point is one sample (*n* = 82). **B** Mean relative abundances (*Z* transformed) of vOTUs significantly differentially abundant by depth (adjusted *p* < 0.05, Likelihood ratio test). Groups were identified through hierarchical clustering and are colored according to the depths in panel **A**. **C** Percentage of vOTUs classified as “aquatic-like” in each of the groups identified in panel B (Groups 1–3) and in the whole dataset of 2699 vOTUs (Total). SPRUCE vOTUs were considered “aquatic-like” if they shared a genus-level viral cluster (VC) with at least one vOTU from a marine or freshwater habitat in the PIGEON database. Note that the y-axis maximum is 10%. *** Denotes a significantly larger proportion of aquatic-like vOTUs in that group, relative to the proportion of aquatic-like vOTUs in the full SPRUCE dataset (Total) (*p* < 0.05, Hypergeometric test)

To characterize peat viral community compositional patterns and their potential drivers, vOTU abundances from the 82 SPRUCE experiment metagenomes were compared with the environmental measurements. Using the 4326 SPRUCE vOTUs as references, we recovered 2699 vOTUs from the SPRUCE experimental plots through read recruitment and tracked their abundances (average per bp coverage depth) across the experimental plot metagenomes. No significant differences in viral community composition were detected according to temperature treatment (Mantel *p* = 0.0057, ρ = 0.56), as discussed in more detail below. Viral community composition was significantly correlated with depth (Fig. 1A), even across different temperature treatments and years (Mantel *p* = 0.57, ρ = 0.00001), consistent with previous evidence that viral community composition varies with depth in Swedish peatlands [15] and other soils [67]. These results are also consistent with observations of microbial communities in SPRUCE peat, where depth explained the largest amount of variation in peat microbial community composition, and temperature effects have thus far (from 2015 to 2018) not been significant [1, 57]. We also measured a significant difference in viral community composition between the two sampling years (June 2015 and June 2016, PERMANOVA *p* = 0.009). Other factors that significantly (*p* < 0.05) correlated with viral community composition included microbial community composition, porewater CO_2_ and CH_4_ concentrations, and the calculated fractionation factor for carbon in porewater δ^13^CH_4_ relative to δ^13^CO_2_ (αC) [68] (Table S3), which can be used to infer CH_4_ production and consumption pathways [3, 15, 68, 69]. Although all of these factors also co-varied with depth, interestingly, viral community composition was more significantly correlated with αC and porewater CH_4_ concentrations than with depth. Together, these results prompted further exploration of potential explanations for these compositional patterns with depth, including links between SPRUCE vOTUs and water content, peat C cycling, and microbial hosts.

To investigate potential drivers of viral community compositional patterns with depth, we identified 121 vOTUs that exhibited significant differential abundance patterns across peat depth levels (adjusted *p* < 0.05, Likelihood ratio test). We assigned these vOTUs to one of three groups via hierarchical clustering (Fig. 1B): vOTUs abundant in the near-surface (10–20 cm) but depleted at other depths, vOTUs abundant from 40 to 50 cm but depleted at other depths, and vOTUs abundant in only the two deepest depth ranges (100–125 and 150–175 cm). Given that near-surface peat had significantly higher gravimetric soil moisture measurements than deeper peat (*p* = 0.002, Student’s *T* test), we used a trait-based approach to assign an “aquatic-like” trait to vOTUs that were found in the same viral clusters (VCs, based on predicted protein content) as vOTUs from freshwater and/or marine environments in our PIGEON database, and then we compared the proportion of aquatic-like vOTUs in the three depth-range groups. Near-surface depths displayed the highest proportion of aquatic-like vOTUs, followed by mid-depths, while the deepest peat had zero recognizable aquatic-like vOTUs (Fig. 1C). The proportion of aquatic-like vOTUs in the near-surface group was significantly higher than the aquatic-like proportion of the total set of 2699 vOTUs (*p* < 0.05, Hypergeometric test), suggesting that vOTUs in the surface horizons (and/or their hosts) might be better adapted to water-rich environments. Consistent with this interpretation, we did not exclude porewater from our samples [3, 7, 15, 44], so it is likely that some of the vOTUs were derived from the porewater directly. Also, although water table depth measurements indicated that the entire sampled peat column was saturated for each of the samples, qualitatively, there was substantially more volumetric water content (waterlogging) in the near-surface depths compared with the deeper, more compacted peat. Although peat viral community composition was significantly correlated with both depth and measured soil moisture content (Mantel *p* < 1E–5), the Mantel *r* value was higher for the correlation with depth (*r* = 0.569) than with soil moisture (*r* = 0.298, Table S3), suggesting that differences in aquatic-like vOTUs alone do not fully explain the patterns in viral community composition with depth. Indeed, the underlying explanation for the observed enrichment of aquatic-like vOTUs in the near surface could be due to a variety of ecological similarities between near-surface peatlands and aqueous systems beyond simply water content (*e.g.*, redox chemistry, substrates, and dissolved oxygen content [42, 70]) and warrants further exploration in the future.

**Fig. 2:**
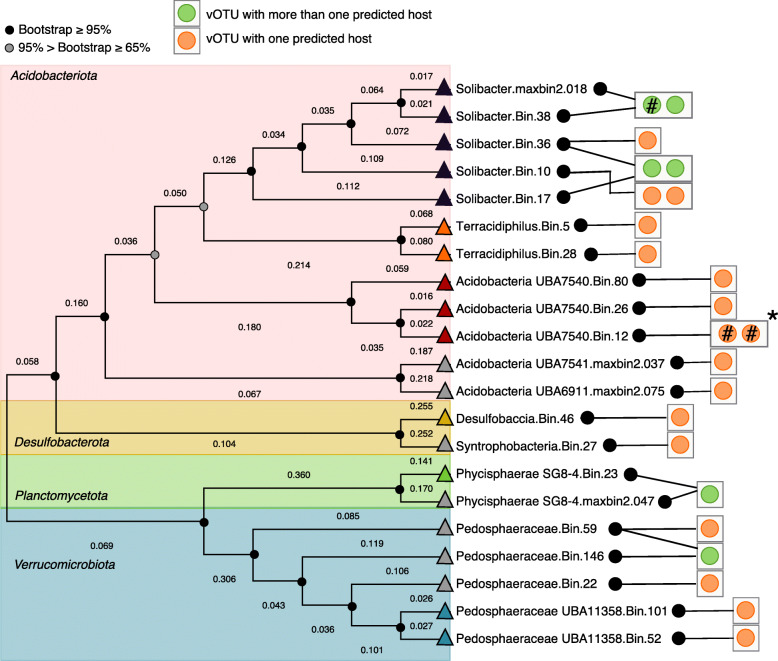
SPRUCE virus–host linkages according to host phylogeny. Unrooted phylogenetic tree (concatenated predicted protein alignment of 43 marker genes defined by CheckM [72]) of microbial host metagenome-assembled genomes (MAGs) with at least one vOTU (green and orange circles) linked via CRISPR sequence homology. Branch lengths represent the expected number of substitutions per site. Lines between black circles and squares with orange or green circles link vOTUs to predicted host MAGs. Colored triangles indicate the MAG genus (the same color is the same genus, except for grey triangles, for which the corresponding MAG could only be classified to the family level). Asterisk indicates vOTUs in the same genus-level viral cluster (VC); remaining vOTUs were all in distinct VCs. Bootstrap support values are shown as circles on nodes, black circles indicate support ≥ 95%, grey indicates support between 65 and 95%. A pound sign inside an orange or green circle indicates a one-nucleotide CRISPR spacer-protospacer mismatch

Under the assumption that patterns in viral community composition were at least partially indirect, resulting from interactions with hosts, we attempted to bioinformatically link SPRUCE vOTUs to microbial host populations [15]. All 4326 vOTUs and a total of 486 bacterial and archaeal metagenome-assembled genomes (MAGs, 443 from the SPRUCE experiment metagenomes (Table S4) and 43 from the transect (> 60% complete, < 10% contaminated, Table S5)) were considered in this analysis. A total of 2870 CRISPR arrays were recovered from the metagenomes via Crass [71], and 29 CRISPR-derived virus–host linkages were made between 23 vOTUs and 21 host MAGs (Fig. 2, Table S6). For 25 of the 29 linkages, 0 mismatches were found between the CRISPR spacers and linked viral protospacers, and four linkages had a one-nucleotide mismatch. All 21 of the MAGs were bacterial and could be taxonomically classified to at least the family level, and for each of the six vOTUs linked to more than one host, the predicted hosts were all in the same family. Where genus-level host classification was possible, all vOTUs were predicted to infect the same host genus.

To investigate potential connections between virus–host dynamics and environmental conditions, along with viral community links to carbon chemistry, we attempted to assess virus–host abundance ratios and their patterns across samples, and we explored the auxiliary metabolic gene (AMG) content of the vOTUs. Only 10 virus–host pairs (10 vOTUs linked to 9 MAGs) were identified for which both the vOTU and the MAG were detected together in at least one sample, and significant patterns in virus–host abundance were not found for any of these pairs according to any of the parameters considered, including depth, year, αC, CH_4_ and CO_2_ concentrations, and moisture content. To further investigate the significant correlation between αC and viral community composition, we also looked for vOTU linkages to methanogen or methanotroph MAGs. HMM searches for McrA (a methanogenesis biomarker) [73, 74], sMMO, pMMO, and pXMO (methanotrophy biomarkers) [3] predicted proteins were performed on the 443 SPRUCE experiment MAGs. Nine MAGs were found to contain McrA-encoding genes, and evidence for methanotrophy was found in 22 MAGs, but none of these MAGs had a CRISPR linkage to a vOTU. Thus, we infer either that αC co-varies with an unmeasured variable that better explains viral community composition and/or that important virus–host linkages associated with CH_4_ cycling were not identified through these approaches. Finally, consistent with potential viral roles in the soil C cycle, we identified 287 putative AMGs encoded by viral genomes predicted to be involved in 18 C cycling processes, based on VIBRANT and DRAM-v output [40, 41] (Table S7, S8, S9). These results are consistent with previously identified glycosyl hydrolase genes encoded in peat viral genomes [13, 15], along with other putative C cycling AMGs from soil [75, 76] (see Supplementary Discussion).

As indicated above, no significant influence of temperature on viral community composition was detected over the first 2 years of experimental warming. Consistent with these findings, no differences in microbial community composition were found according to temperature treatments in these samples over the first 5 years of whole-ecosystem warming, although warming exponentially increased CH_4_ emissions and enhanced CH_4_ production rates throughout the entire soil profile [57]. These results are also consistent with prior studies that have shown that soil microbial community responses to similar temperature increases can take multiple years to manifest [77–79]. Warming has been shown to substantially alter the community composition, diversity, and N_2_ fixation activity of peat moss microbiomes [58], and in microcosms of surface peat collected from the SPRUCE site, microbial diversity was negatively correlated with temperature, suggesting that prolonged exposure of the peatland ecosystem to elevated temperatures will lead to a loss in microbial diversity [80]. In the SPRUCE experiment, the fractional cover of *Sphagnum* mosses [46] and plant phenology (the timing of different traits throughout the growing season) [54] have changed in response to temperature, suggesting that differences in belowground viral and microbial community composition may follow after a longer period of warming.

### Placing SPRUCE peat viruses in global and ecosystem context

**Fig. 3:**
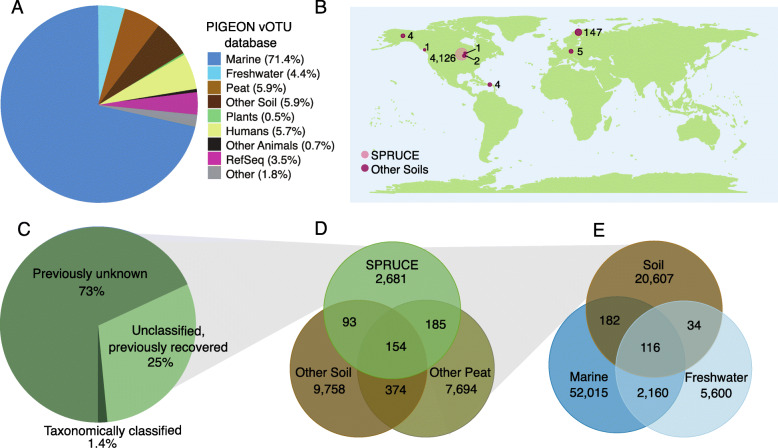
Habitat and global distribution of SPRUCE vOTUs and viral clusters (VCs), using the PIGEON database for context. **A** Composition of the PIGEON database of vOTUs (*n* = 266,805) by source environment. RefSeq includes isolate viral genomes from a variety of source environments (prokaryotic viruses in RefSeq v95). Plants = plant-associated, Humans = human-associated, Other Animals = non-human animal-associated. **B** vOTUs (*n* = 4326) recovered from SPRUCE peat by read mapping, according to the location from which they were first recovered. Numbers indicate SPRUCE vOTUs from a given location. Circle sizes are proportional to the number of vOTUs. **C** Percentages of vOTUs recovered from SPRUCE that had predicted taxonomy based on clustering with RefSeq viral genomes (Taxonomically classified), had unknown taxonomy but shared a genus-level viral cluster (VC) with one or more previously recovered vOTUs in the PIGEON database (Unclassified, previously recovered), or were previously unknown at the VC (genus) level (Previously unknown). **D** Habitat(s) for each soil VC (*n* = 20,939) in the PIGEON database, based on source habitat(s) for the vOTU(s) contained in each VC. For a given soil VC, either all vOTUs were exclusively derived from a single habitat (non-overlapping regions), or two or more vOTUs were derived from different soil habitats (overlapping regions). **E** Similar to **D**, but for VCs with vOTUs from soil, marine, and/or freshwater habitats (*n* = 80,714 VCs)

Of the 4326 “species-level” vOTUs from SPRUCE, 4162 were assembled from SPRUCE-associated metagenomes (including the viromes), and 164 were recovered through read mapping to our PIGEON database of vOTUs from diverse ecosystems (Fig. 3A). The 164 previously recovered vOTUs were first reported from other globally distributed sites, mainly peatlands (160 of 164), including peat vOTUs from Sweden (147), Germany (5), Alaska, USA (4), Wisconsin, USA (2), and Canada (2) (Fig. 3B). The recovery of hundreds of viral species (4% of the dataset) in geographically distant peatlands suggests that there may be a peat-specific niche for these viruses. In addition, four vOTUs recovered from SPRUCE peat were first identified in a wet tropical soil in Puerto Rico, suggesting some global species-level sequence conservation across soil habitats (Table S10). Existing deeply sequenced soil viromic datasets are predominantly from peat [7, 13, 15, 34], so the extent to which these patterns reflect database bias or true differences between peat and other soils will require additional sampling.

Interestingly, despite the overwhelming dominance of marine vOTUs in our database (190,502 vOTUs, 71%), zero species-level vOTUs from the oceans were recovered in the SPRUCE peatlands. Freshwater vOTUs (predominantly from freshwater lakes) have less representation in our database (11,869 vOTUs, 4.45%), but similarly, no freshwater vOTUs were recovered from SPRUCE peat (though, as described above, vOTUs that shared higher-level taxonomy with aquatic viruses were recovered in SPRUCE peatlands). No other vOTUs from our PIGEON database, including bioreactor, hot spring, non-peat wetland, human-, plant-, and other host-associated vOTUs, were recovered in SPRUCE peat. These results suggest viral adaptation to soil and/or strong viral species boundaries between terrestrial, aquatic, and other ecosystems, as previously observed for bacterial species [81, 82], though data for soil viruses are limited, so further studies across diverse soils will be necessary to assess the generalizability of these results.

**Fig. 4:**
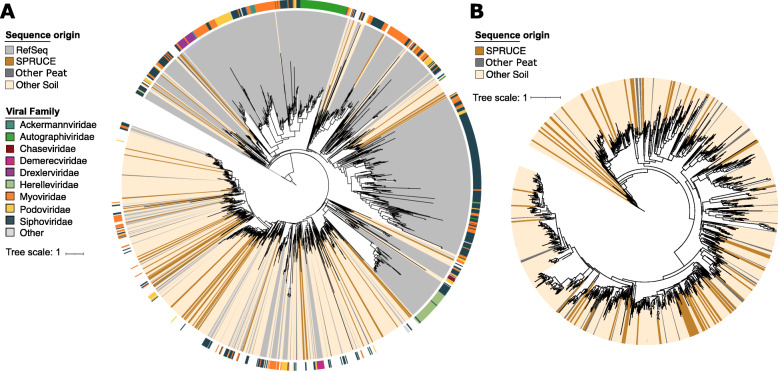
Unrooted phylogenetic trees of terminase large subunit (TerL) protein sequences from RefSeq prokaryotic viral genomes and soil vOTUs in the PIGEON database. Trees are color-coded by sequence source (RefSeq or soil category within PIGEON). Trees were constructed using IQ-tree and the LG+I+G4+F model of sequence evolution, using ultrafast bootstrapping and an SH-aLRT test. Bootstrap values are not displayed but can be found for each of the branches in Supplemental File 1. **A** Phylogenetic tree of TerL protein sequences from RefSeq prokaryotic viral genomes (*n* = 1613) and PIGEON soil vOTUs (*n* = 1011). Outer ring color represents viral family of RefSeq genomes. Phylogenetic dispersion was estimated by using Fritz & Purvis D (*D*). *D* = − 0.25 when comparing TerL sequences from RefSeq viral genomes and TerL sequences from soil vOTUs, with *D* < 0 indicating phylogenetic clustering. **B** Phylogenetic tree of TerL protein sequences from PIGEON soil vOTUs. *D* = 0.58 for other soil (*n* = 634) compared with peat, including SPRUCE (*n* = 377), and *D* = 0.41 when comparing SPRUCE (*n* = 51) to all other soil sequences (*n* = 960)

To further compare vOTUs from diverse soil ecosystems, we constructed a phylogenetic tree of the terminase large subunit (terL) gene from 1045 PIGEON soil vOTUs (81 from SPRUCE, 143 from other peat, and 821 from other soil) and 1613 RefSeq prokaryotic viral genomes from which a terL sequence could be recovered (Fig. 4A). The terL gene is a single-copy viral marker gene [12] that is commonly used for phylogenetic tree construction of *Caudovirales* phages [83, 84], due to its ubiquity and relatively high sequence conservation across diverse phages [84]. Overall, the tree revealed two large superclades, one with predominantly RefSeq viral sequences and one with predominantly soil viral sequences (phylogenetic dispersion, *D* = − 0.25), with *D* < 0 indicating significant phylogenetic separation of RefSeq and soil sequences [85, 86]. As expected, these results indicate that known isolates do not adequately capture soil viral diversity. A second terL tree was constructed from only the soil sequences without RefSeq (Fig. 4B), revealing approximately even phylogenetic distributions across soil habitats and no detectable soil habitat-specific phylogenetic groupings (*D* = 0.58 for all peat vs. other soil, *D* = 0.41 for SPRUCE vs. all other soil). In other words, phylogenetically similar viruses (at least based on terL phylogeny) were found across the three examined soil habitat groupings (SPRUCE, other peat, and other soil), with no significant differences in viral types recovered across these groups or when comparing all peat viruses to those from other soil.

To assign taxonomy to vOTUs and group them at higher taxonomic levels for cross-ecosystem comparisons, the 4326 SPRUCE vOTUs and all other vOTUs in our PIGEON database were grouped into viral clusters (VCs), according to their shared predicted protein content [87, 88]. The SPRUCE vOTUs formed 3114 VCs, 2193 of which were singletons and 921 of which contained at least two vOTUs (Table 1, Supplementary figure 2A). We note that, although singletons are not technically clusters, each VC has been suggested to represent a distinct viral “genus” [87, 88], so we include singletons in VC counts for ease of interpretation. We describe each VC as a “genus”, in accordance with previously described terminology for this approach [87, 88], but viral taxonomy is in flux [89, 90], and an analysis of average amino acid identity (AAI) within 100 randomly chosen PIGEON VCs revealed that most VCs represent the equivalent of bacterial family or higher taxonomy. Briefly, vOTUs within most VCs shared an average of 45–65% AAI (for bacteria, that AAI range approximates the same family but different genera [91]), though ~ 1/3 of the VCs had average AAIs above or below this range. Only fourteen of the SPRUCE VCs, containing 61 vOTUs (1.4% of the dataset), were taxonomically classifiable, based on sharing a VC with a viral genome in RefSeq (Fig. 3C, Supplementary figure 3). This is a lower proportion than a prior study [15], which we attribute at least in part to differences in the size of the dataset used for clustering (for example, 17% of peat vOTUs from northern Sweden were previously taxonomically classifiable [15], but only 3.9% of those same vOTUs could be taxonomically classified in our analysis, which included orders of magnitude more vOTUs but was otherwise similar, apart from use of the updated vConTACT2.0 pipeline instead of vConTACT). The taxonomically classifiable vOTUs from SPRUCE included 45 Myoviridae, five Podoviridae, four Siphoviridae, and seven Tectiviridae, consistent with the more abundant viral taxa previously reported from thawing permafrost peatlands [15], but we note that Myo-, Podo-, and Siphoviridae have been recommended for removal as taxonomic groups [88]. Although most SPRUCE VCs were not taxonomically classifiable, 562 included a vOTU that was also found in another dataset in PIGEON, meaning that just under 1/3 of the SPRUCE VCs had been observed before (compared with previous detection of only 4% of SPRUCE vOTUs, or viral “species”, as described above).

**Table 1:** Number of aquatic and soil vOTUs and VCs in the PIGEON database, according to the source environments considered in this study

Dataset	vOTUs	Total VCs	VCs with > 1 vOTU	Singleton VCs (1 vOTU)	vOTUs in a VC	% Singleton VCs	% vOTUs in Singleton VCs
PIGEON aquatic and soil*	233,420	81,846	29,167	52,679	181,987	64	22
Marine	190,502	54,473	25,116	29,357	161,145	54	15
Freshwater	11,869	7910	3257	4653	7216	59	39
All soil	31,049	20,939	4415	16,524	13,626	79	53
SPRUCE**	4326	3114	921	2193	2133	70	51
Other peat	10,831	8414	1377	7037	3794	84	65
Other soil	15,892	10,391	2117	8274	7618	80	52

For each row, the number of viral populations (vOTUs), viral clusters (VCs) with more than one member, and singletons (both vOTUs and VCs with only one member), along with the corresponding percentages that they represent are presented

*Only marine, freshwater, and soil; not including vOTUs from human, other animal, plants or other systems (total PIGEON vOTUs across all environments = 266,125)

**All vOTUs recovered in the SPRUCE experimental plots and transect, including 160 vOTUs also recovered in other peat and 4 vOTUs also recovered in other soil

All 31,049 of the vOTUs from soil in our PIGEON database, including those from SPRUCE and globally distributed soils, grouped into 20,939 VCs (Table 1). Of these, 16,524 included only a single vOTU, meaning that most of the known “genus-level” soil viral sequences have only been recovered from a single study and/or location so far. In total, 12.8% of the soil VCs were exclusively found in SPRUCE peatlands, 0.7% included at least one vOTU each from SPRUCE, other peat habitats, and other soils (Fig. 3D), and 0.9% contained a vOTU from SPRUCE and other peat sites but not other soils. Together, these data suggest that, although much of soil viral sequence space remains to be explored, species-level similarities may be relatively restricted to specific soil habitat types, while similarities at higher taxonomic levels may be more common across soil habitats.

To investigate similarities between viruses from soil and aquatic (marine and freshwater) ecosystems, 233,420 vOTUs from our PIGEON database (31,049 soil [10, 15, 31, 35], 190,502 marine [31, 63, 64], and 11,869 freshwater [31]) were clustered into 80,714 VCs (Table S11). Of the soil VCs, 0.4% shared a cluster with vOTUs from one or both aquatic systems, indicating a small amount of “genus-level” similarity between aquatic and soil viruses (Fig. 3E). However, most VCs were found in only one habitat, consistent with differences in microbial community composition in aquatic compared with soil and sediment habitats and between freshwater and saltwater environments [81].

### Comparing viral recovery from viromes and total soil metagenomes

**Fig. 5:**
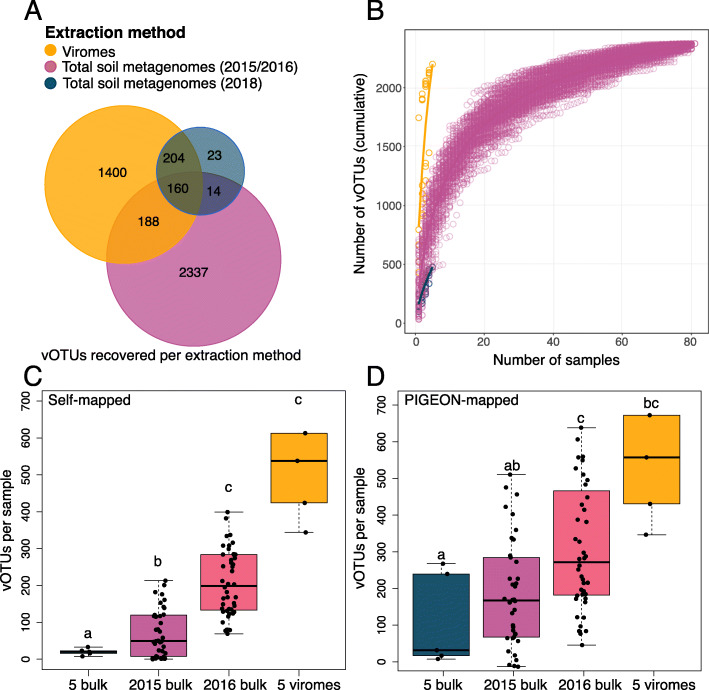
Comparison of vOTU recovery from SPRUCE viromes and total soil metagenomes. **A** Distribution of vOTUs recovered in each of three extraction groups (grouped by extraction method and collection date), based on read mapping to the PIGEON database (*n* = 5 viromes from 2018, 82 total soil metagenomes from 2015 to 2016, and 5 total soil metagenomes from 2018). **B** Accumulation curves of distinct vOTUs recovered as sampling increases for each extraction method; 100 permutations of sample order are depicted as open circles, line shows the average of the permutations for each method. **C** Number of vOTUs recovered per metagenome when reads were only allowed to map to vOTUs that assembled from metagenomes in the same category (self-mapped), considering four categories: 2018 bulk (*n* = 5), 2015 bulk (*n* = 38), 2016 bulk (*n* = 44), 2018 viromes (*n* = 5); bulk = total soil metagenomes. One outlier was excluded from the plot for ease of visualization; the y-axis value of the outlier in the 2018 viromes was 1328. Letters above boxes correspond to significant differences between groups (Student’s *T* test, significant when *p* < 0.05). **D** Similar to **C**, but reads were allowed to map to all vOTUs in the PIGEON database (PIGEON-mapped), including all vOTUs assembled from any of the SPRUCE metagenomes. Three outliers were removed from the plot for ease of visualization; the y-axis values of the two outliers from 2016 bulk were 1415 and 1818, and the value of the outlier from the 2018 viromes was 1558

Metagenomic studies of viral community composition typically take one of two approaches: either the viral signal is mined from total metagenomic assemblies, which predominantly tend to contain bacterial sequencing data [13, 15, 31], or viral particles are physically separated from other microbes in the laboratory (*e.g*., through filtration), and then viral size-fraction enriched metagenomes (viromes) are sequenced and analyzed [12, 13, 15, 18]. To directly compare results from both approaches, we first analyzed the paired total soil metagenomes and viromes from the five transect samples. Considering all assembled contigs ≥ 10 kbp, only 0.8% of the metagenomic contigs were classified as viral after passing them through viral prediction software (see “Methods”), relative to 16% of the virome contigs. This ~ 20-fold improvement is consistent with our observed ~ 30-fold improvement in viral contig recovery from viromes relative to total metagenomes in agricultural soils [35], and similar differences in the composition of metagenomes and viromes have been reported from grassland soils [92]. When accounting for read mapping to all vOTUs in the PIGEON database (including all of the SPRUCE vOTUs), 1952 vOTUs were detected in the viromes, relative to 401 in the metagenomes from the same samples (Fig. 5A, Supplementary figure 4A). Only 37 vOTUs were detected in the metagenomes alone. Although far more vOTUs were recovered from the viromes, vOTU accumulation curves were still climbing steeply after five samples for both viromes and metagenomes (Fig. 5B, Supplementary figure 4B, 4C), suggesting that more viral diversity remains to be recovered. A comparison of the five viromes indicated that there was no spatial relationship between the samples (Supplementary figure 5A), but there was high variability in the number of recovered vOTUs per sample (Supplementary figure 5B).

To place these comparisons from the same samples in the context of the larger SPRUCE dataset, we compared the five viromes from 2018 with the 82 metagenomes from 2015 to 2016, again with vOTU recovery assessed through read recruitment to all vOTUs in the PIGEON database. We note that the samples in this set of comparisons differ in multiple ways beyond the extraction method, including the sampling year, depth range, location, and (in some cases) temperature treatment, all of which could contribute to the observed trends. On a per-sample basis, the viromes recovered far more vOTUs than the metagenomes, as indicated by the much steeper accumulation curve slope for viromes after only five samples (Fig. 5B). However, the much larger number of samples in the SPRUCE experimental plot metagenomes resulted in a higher total vOTU recovery of 2699 in the 82 metagenomes, compared with 1952 in the five viromes (Fig. 5A).

We next considered the metagenomes from 2015 and 2016 separately, because the sequencing throughput from 2016 was 1.4 times higher than in 2015. The first of these comparisons was based on read recruitment only to vOTUs derived from contigs that assembled from samples in the same category, considering four categories: the five transect viromes, five transect metagenomes, 38 metagenomes from 2015, and 44 metagenomes from 2016. These “self-mapped” analyses were meant to simulate a situation in which only the vOTUs from that particular dataset would have been available. The perceived viral richness per sample was 32 times higher in viromes (mean 649 vOTUs) compared with their paired metagenomes (mean 20 vOTUs) but was nine and three times higher, respectively, in viromes compared with the 2015 and 2016 metagenomes (mean 72 and 207 vOTUs) (Fig. 5C). The perceived viral richness was 2.8 times higher in the 2016 metagenomes compared with 2015 metagenomes, indicating that a greater sequencing depth of total soil metagenomes (in this case from 6 to 15 Gbp on average) likely increased vOTU recovery, though we cannot exclude the possibility of a true difference in viral richness between the 2 years. A further comparison of vOTU recovery from the transect viromes and the three sets of metagenomes was based on read recruitment to all 266,125 PIGEON vOTUs from SPRUCE and other datasets. In this case, the perceived viral richness in the viromes (mean 721 vOTUs) was 5.7 times higher than in the paired metagenomes (mean 127 vOTUs), 3.5 times higher than in the 2015 metagenomes (mean 200 vOTUs), and two times higher than in the 2016 metagenomes (mean 370 vOTUs, Fig. 5D). Thus, the availability of reference vOTUs, particularly from the SPRUCE viromes, substantially improved recovery from the total metagenomes.

Lastly, we compared the VCs formed by vOTUs from the 2018 viromes, the 2018 metagenomes, and the 2015/2016 metagenomes to determine whether there were differences in the taxonomic space recovered by the different approaches. When comparing the five paired total metagenomes and viromes, all of the metagenome vOTUs shared a VC with at least one vOTU from the viromes, whereas 1401 vOTUs were in VCs exclusively recovered from the viromes, indicating that viromes expanded the recoverable viral taxonomic space relative to paired metagenomes (Supplementary figure 2A, 2B). However, the vOTUs recovered from the unpaired 2015/2016 metagenomes recovered substantially different VCs compared with the 2018 viromes. We suspect that these differences were largely due to the different collection years, locations, and, particularly, numbers of samples, as opposed to differences between extraction methods.

Few direct comparisons of viromes and total metagenomes from the same samples have been reported from any ecosystem. Consistent with these results from peat, agricultural and grassland soil viromes have been shown to be enriched in both viral sequences and genomes from ultrasmall cellular organisms (which would be more likely to pass through the 0.2 μm filters used for viral enrichment) but depleted in sequences from most other cellular organisms, compared with total metagenomes [35, 92]. In aqueous systems, water samples are often separated into multiple-size fractions (for example, 3–20 μm, 0.8–3 μm, 0.2–0.8 μm, post-0.2 μm), such that previous studies have compared viral sequences recovered across different size fractions, and generally, the viruses recovered from different size fractions seem to be distinct [93, 94]. A recent meta-analysis of human gut viral data recovered from viromic and metagenomic sequences suggested that more viral contigs could be recovered from metagenomes than from viromes [90]. However, of the 2017 viromes considered in that study, 1966 were multiple-displacement amplification (MDA) treated, and, as the authors acknowledged, MDA of viromes has known methodological biases (for example, MDA preferentially recovers circular ssDNA viruses [6]) and thus would result in artificially lower-richness viral communities. Although differences in the environments could have contributed to the observed differences in viral recovery from viromes compared with total metagenomes in the human gut study compared with our work, the large difference in the number of total metagenomes (680) compared with non-MDA amplified viromes (51) in the human gut study could also have contributed to the greater recovery of viral sequences from total metagenomes in that study. Consistent with that interpretation, here we have shown that increasing the number of samples, in combination with deeper sequencing and the availability of relevant reference vOTU sequences, improved vOTU recovery from total soil metagenomes, which have the added advantage of accessing virus and host population sequences from the same dataset.

## Conclusions

We analyzed dsDNA viral diversity in a climate-vulnerable peat bog, revealing significant differences in viral community composition at different soil depths and according to peat and porewater C chemistry. Aquatic-like SPRUCE vOTUs were significantly more abundant at near-surface depths, suggesting potential adaptation of these viruses to water-rich environments. Some viral species-level similarities were observed across large geographic distances in soil: 4% of the vOTUs found in SPRUCE peat were previously recovered elsewhere, predominantly in other peatlands. Interestingly, zero marine or freshwater vOTUs were recovered from SPRUCE peat, suggesting the potential for viral species boundaries between terrestrial and aquatic ecosystems. When comparing vOTU recovery from viromes and total soil metagenomes, increasing the dataset size through deeper sequencing and more samples improved vOTU recovery from metagenomes, but viromics was a better approach for maximizing viral recovery on a per-sample basis. Together, these results expand our understanding of soil viral communities and the global soil virosphere, while hinting at a vast diversity of soil viruses remaining to be discovered.

## Materials and methods

### Sample collection

In June 2018, five peat samples were collected along “Transect 4” in the S1 bog ~ 150 m from the SPRUCE experimental plots in the Marcell Experimental Forest in northern Minnesota, USA (for GPS coordinates, see Table S12). Avoiding green *Sphagnum* moss at the surface (~ 2 cm), the top 10 cm of peat (5 cm diameter) was collected for each sample with a sterile spatula and placed in 50-mL conical tubes on dry ice. Samples were stored at − 80 °C for 6 months prior to DNA extraction for total metagenomes and viromes.

Within the SPRUCE study, temperature treatments were applied in large (~ 115 m^2^) open-topped enclosures. Temperature treatments in the 10 enclosures were as follows: + 0, + 2.25, + 4.5, + 6.75, and + 9, with two chambers assigned to each temperature treatment. Data were also collected from two ambient environment plots where there was no enclosure but within the treatment area on the south end of the S1 Bog. In each enclosure, warming of deep soil started in June 2014 [47], and aboveground warming began in August 2015 with continuous whole ecosystem warming (365 days per year) operating since late in 2015. A more detailed explanation of deep soil heating procedures and construction of the enclosures and warming mechanics can be found in Hanson et al. [46, 47, 54].

Peat samples for 82 total soil metagenomes were collected from the SPRUCE experiment in June 2015 and June 2016 from cores that were extracted using defined hand sampling near the surface and via Russian corers below 30 cm. Samples for analysis were obtained from depth ranges 10–20 cm, 40–50 cm, 100–125 cm, and 150–175 cm from a total of 10 chambers in 2015 (no samples were analyzed from the open, ambient plots that year), with the exception of only two samples collected from chamber 19 (control plot, no temperature treatment, only 10–20 cm and 40–50 cm samples collected), for a total of 38 samples from 2015. In 2016, samples were collected from the same depth ranges from all 10 chambers, plus two samples from each of the two ambient, open plots (depth ranges, 10–20 cm and 40–50 cm), for a total of 44 samples from 2016. These 82 samples were used for DNA extraction and total metagenomic analysis and MAG recovery, as described below. Soil temperature, moisture content, CH_4_ and CO_2_ concentrations, and a_C_ measurements (see supplementary methods) were collected from the same samples (Table S13).

### DNA extraction

All samples from the peatland transect were stored at – 80 °C until further processing. Twenty-four hours prior to DNA extraction, samples were placed at − 20 °C. For total metagenomes from the transect, DNA was extracted from 0.25 g peat per sample with the QIAGEN DNeasy Powersoil Kit (QIAGEN, Germany), according to the manufacturer’s protocol. For viromes, 50 g of peat per sample was divided between two 50-mL conical tubes, and 37.5 mL of Amended Potassium Citrate Prime buffer (AKC’, 0.02 μm filtered, 1% K-citrate + 10% PBS + 150 mM MgSO_4_) [34] was added per tube, for a total of 75 mL buffer. Tubes were shaken at 400 rpm for 15 min, then centrifuged at 4700 g for 20 min. Excluding the pelleted soil, the supernatant was filtered through a 0.2 μm polyethersulfone filter (Corning, USA) and ultracentrifuged in a Beckman LE-8K ultracentrifuge with a 70 Ti rotor for 3 h at 32,000 RPM at 4 °C under vacuum. The supernatant was decanted, and the pellet containing virions was resuspended in 200 μl UltraPure water and added to the QIAGEN DNeasy PowerSoil Kit bead tubes (QIAGEN, Germany) for DNA extraction according to the manufacturer’s instructions with one exception: instead of vortexing for 10 minutes with the beads, samples in the bead tubes were incubated at 70 °C for 10 min, vortexed briefly, and incubated at 70 °C for another 5 min. Consistent with our prior work on hypersaline lake viromes, which showed that DNase treatment of viromes stored frozen resulted in removal of all DNA [95], and given recent evidence for the same ecological patterns preserved in data from both DNase treated and untreated viromes from the same agricultural soil samples [96], we elected not to include a DNase treatment prior to virion lysis.

For the 82 2015 and 2016 peat samples used in metagenomic analysis and MAG recovery, DNA was extracted from homogenized samples of each depth interval using the MO BIO Powersoil DNA extraction kit (QIAGEN, Germany). Six replicate 0.35-g extractions were combined and re-purified with the MO BIO PowerClean Pro kit (QIAGEN, Germany) and eluted in 50 mL of 10 mM Tris buffer.

### Library construction and sequencing

Library construction and sequencing for the five viromes and five total soil metagenomes from Transect 4 were conducted by the DNA Technologies and Expression Analysis Cores at the UC Davis Genome Center. Libraries were prepared with the DNA Hyper Prep library kit (Kapa Biosystems-Roche, Basel, Switzerland), as previously described [35]. There was no whole-genome amplification or equivalent; standard metagenomic library construction was applied directly to extracted DNA for both the viromes and total metagenomes. Paired-end sequencing (150 bp) was done on the Illumina NovaSeq platform, using 4% of a lane per virome and 8% of a lane per total soil metagenome. Sequencing of the 82 metagenomes from the SPRUCE experiment and ambient plots was done by the DOE Joint Genome Institute (JGI), using standard protocols for Nextera XT metagenomic library construction. These barcoded libraries were sequenced on an Illumina HiSeq 2500 instrument in 2x150 bp mode.

### Sequencing read processing, assembly, viral population (vOTU) recovery, and read mapping

Raw reads from the SPRUCE experiment metagenomes (82), transect viromes (5), and transect total soil metagenomes (5) were first quality-trimmed with Trimmomatic v0.38 [97] with a minimum base quality threshold of 30 evaluated on sliding windows of 4 bases and minimum read length of 50. Reads mapped to the PhiX genome were removed with bbduk [98]. Reads were assembled into contigs ≥ 10 kbp in length, using MEGAHIT v 1.1.3 [99] with standard settings. All 92 metagenomes underwent single-sample assemblies, and two additional co-assemblies were generated from the transect, one each for the five viromes and five total soil metagenomes, respectively. For co-assemblies, the preset meta-large option was used. Eighty-two previously existing assemblies from the SPRUCE experiment metagenomes were also used. Briefly, for those assemblies, raw metagenomic fastq sequences were quality trimmed with bbduk from the BBTools software package (options: qtrim=window,2 trimq=17 minlength=100) [100] and assembled with IDBA-UD [101](options: -mink 43 –maxk 123 –step 4 –min_contig 300).

DeepVirFinder [39] and VirSorter [38] were used to recover viral contigs from each assembly. VIBRANT [40], which we used for auxiliary metabolic gene (AMG) analyses described below, was not available at the time that these viral prediction analyses were performed. Briefly, DeepVirFinder is a machine-learning approach that recognizes viral sequence signatures, and VirSorter searches for viral hallmark genes in PFAM annotation. Consistent with established recommendations, contigs with DeepVirFinder scores > 0.9 and *p* < 0.05 were considered viral [64], and DeepVirFinder results were filtered with a custom python script (parse_dvf_results.py, all scripts are available on GitHub, see Data Availability Statement below) to only retain results in compliance with this score. VirSorter was run in regular mode for all total metagenomes and in virome decontamination mode for the viromes. Only contigs from VirSorter categories 1, 2, 4, and 5 (high-confidence) were retained, as previously recommended [38]. All resulting viral contigs were clustered into vOTUs using CD-HIT [102] at a global identity threshold of 0.95 across 85% of the length of the shorter contig [61]. Different sets of vOTUs were used as references for read mapping throughout the manuscript (see main text), with the most commonly used and most comprehensive reference database being PIGEON (see below). In all cases, read mapping was performed with BBMap [98] at ≥ 90% identity, following thresholds set previously [15, 61, 103], and vOTU coverage tables were generated with BamM [104], using the ‘tpmean’ setting, and bedfiles were generated using bedtools [105]. Custom python scripts (percentage_coverage.py, filter_coveragetable.py) were used to implement the thresholds for detecting viral populations (vOTUs) in accordance with community standards (≥ 75% of the contig length covered ≥ 1× by reads recruited at ≥ 90% nucleotide identity) [61]. The final vOTU coverage table of per-bp vOTU abundances in each metagenome was normalized by the number of metagenomic sequencing reads for each sample [15].

### Construction of the PIGEON reference database of vOTUs

An in-house database, *P*hages and *I*ntegrated *G*enomes *E*ncapsidated *O*r *N*ot (PIGEON), was created, containing 266,125 species-level vOTUs, of which 190,502 came from marine environments, 11,869 from freshwater, 31,049 from soil (including 4326 from SPRUCE), 2305 RefSeq viral genomes (release 85) [65], and 30,400 from other environments in a meta-analysis, including human microbiomes, other animal microbiomes, plant microbiomes, and other environments). Available viral contigs were downloaded from published datasets [10, 13, 15, 31, 34, 62–66], compiled from ongoing work in Alaskan peat soil and Puerto Rican soils (see supplementary methods), and those recovered from SPRUCE (see above). For most of the previously published datasets, viral contigs were derived from viromes, or a combination of viromes and total soil metagenomes, but two datasets only considered viral recovery from total soil metagenomes [10, 31]. For all but one of the datasets, VirSorter [38], VirFinder [106], DeepVirFinder [39], or a combination of these programs was used for viral contig recovery (Contigs with DeepVirFinder scores > 0.9 and *p* < 0.05 were considered viral [64], and only contigs from VirSorter categories 1, 2, 4, and 5 were considered). The exception was the meta-analysis dataset of Paez-Espino et al., which used its own viral discovery pipeline [31]. From all of these datasets, viral contigs were downloaded, and those > 10 kbp were retained and then clustered into vOTUs using CD-HIT [102] at a global identity threshold of 0.95 across 85% of the shorter contig length to generate PIGEON v1.0. We are actively improving PIGEON and expect to release a new version in the future.

### Viral taxonomic classification and construction of viral clusters (VCs) through protein-based clustering of vOTUs

VCs were generated to perform analyses at higher taxonomic levels than ‘species’, and taxonomic classifications for the 4326 SPRUCE vOTUs (detected in the SPRUCE dataset through read mapping) were assigned at the VC level. To generate VCs and assign taxonomy, the vOTUs were clustered according to shared predicted protein content with the 261,799 other vOTUs in our PIGEON database, including 2305 RefSeq viral genomes [65], using vConTACT2 (options: --rel-mode ‘Diamond’ --db 'ProkaryoticViralRefSeq85-Merged' -pcs-mode MCL --vcs-mode ClusterONE) [87, 88]. Taxonomy was assigned by vConTACT2 to any vOTU that shared a VC with one or more RefSeq viral genomes, as previously described [87, 88]. The vConTACT2 viral_cluster_overview output file was used for further analysis, including to manually identify SPRUCE vOTUs that shared a VC with one or more vOTUs from marine and/or freshwater (aquatic) environments. For the analysis of AAI within PIGEON VCs, a random set of 100 VCs was analyzed with CompareM (standard settings) [107], and the mean pairwise AAI between vOTUs was calculated for each of those VCs.

### Metagenome-assembled genome (MAG) reconstruction

MAG reconstruction from the five transect total metagenomes was done as follows: quality-trimmed reads were assembled using MEGAHITv 1.1.3 [99] with a minimum contig length of 2000, using the meta-large preset. After individual assembly of each sample, quality-filtered and trimmed reads were mapped to the resulting contigs using bbmap [108] with standard settings, and this abundance information was used to bin the contigs into MAGs using MetaBAT [109], using the --veryspecific setting and the coverage depth information. Quality and identification of bins was done with CheckM [110], following Sorensen et al. [72].

From the 82 SPRUCE experiment metagenomes, metagenome assembly, recovery, and analysis of metagenome-assembled genomes (MAGs) was performed as described in Johnston et al. [111]. Briefly, metagenomic sequences were assembled with IDBA-UD [101] (options: -mink 43 –maxk 123 –step 4 –min_contig 300). Resulting contigs ≥ 2.5 kbp were used to recover microbial population genomes with MetaBAT2 (options: –minCVSum 10) [109] and MaxBin2 [112]. Before binning, Bowtie 2 was used to align short-read sequences to assembled contigs (options: –very-fast) [113], and SAMtools was used to sort and convert SAM files to BAM format [114]. Sorted BAM files were then used to calculate the coverage (mean representation) of each contig in each metagenome. The quality of each resulting MAG was evaluated with the CheckM v1.0.3 taxonomy workflow for Bacteria and Archaea separately [110]. The result from either evaluation (i.e., taxonomy workflow for Archaea or Bacteria) with the highest estimated completeness was retained for each MAG. MAGs with a quality score ≥ 60 were retained (from Parks et al., 2017 [115] calculated as the estimated completeness – 5 × contamination). MAGs recovered from different metagenomes were dereplicated with dREP [116], and the GTDB-tk classify workflow [117, 118] was used to determine MAG taxonomic affiliations. MAG gene prediction, functional annotation, and assessment of metabolic pathway completeness (e.g., for assessing methanogenesis potential) was performed as described in Johnston et al. [111]. Taxonomic classification, source dataset SRA ID, basic genome statistics, and CheckM summaries for each MAG can be found in Table S4.

Using the parameters described above for vOTU coverage table generation, a microbial contig coverage table was generated. From this coverage table, we calculated the coverage of each population genome as the average of all of its binned contig coverages, weighting each contig by its length in base pairs. In-house scripts for this are available on GitHub. HMM searches were done on both MAGs and vOTUs for proteins involved in methanogenesis or methanotrophy (McrA (a methanogenesis biomarker) [73, 74], sMMO, pMMO, and pXMO (methanotrophy biomarkers) [3]). The MAG and vOTU contigs were annotated with prodigal (standard settings) [119], and an HMM search was done on these annotations with hmmr [120], using hmmsearch (standard settings) with an *e* value cutoff of 1E–5 [74].

### Reconstruction of microbial CRISPR arrays and virus–host linkages

CRISPR repeat and spacer arrays were assembled with Crass v0.3.12 [71], using standard settings, and BLASTn was used to match spacer sequences with vOTUs and repeats to MAGs, in order to link viruses to putative hosts. Briefly, for protospacer-spacer matches (*i.e.*, matches between vOTUs and CRISPR spacer sequences), the BLASTn-short function was used, with ≤ 1 mismatch to spacer sequences, *e* value threshold of 1.0 × 10^−10^, and a percent identity of 95 [31, 121]. For MAG-repeat matches, the BLASTn-short function was used, with an *e* value threshold of 1.0 × 10^−10^ and a percent identity of 100 [15].

### Phylogenetic tree construction

A phylogenetic tree of bacterial host MAGs with CRISPR matches to one or more vOTUs (*i.e*., a repeat match to a MAG and a spacer from the same CRISPR array with a match to a vOTU protospacer) was constructed with CheckM [110] via a marker-gene alignment of 43 conserved marker genes with largely congruent phylogenetic histories, defined by CheckM [110]. This alignment was used to construct a maximum-likelihood tree with MEGA [122], with the LG plus frequencies model [123]. A total of 500 bootstrap replicates were conducted under the neighbor-joining method with a Poisson model.

For the terminase large subunit (TerL) tree, we predicted proteins on all viral contigs from PIGEON soil-associated vOTUs (*n*=31,346) with Prokka [124], (std settings, --kingdom viruses, --norrna –notrna), resulting in 1045 large terminase subunit predictions. We downloaded the terminase large subunits (*n* = 2799) that were available from RefSeq and clustered the Refseq terminase sequences at 95% AAI using USEARCH, following [32, 125], resulting in 1613 terminase sequences from RefSeq. We then aligned predicted terminase sequences from PIGEON soil vOTUs with those from RefSeq (2658 sequences total), using MAFFT v7.471 [126] with the G-INS-1 algorithm and otherwise standard settings [32]. Ambiguous aligned regions were removed using the TrimAl v1.41 program with the ‘gappyout’ setting [127, 128]. The best model of amino acid substitution was determined using ProtTest v1.5, standard settings [129]. Phylogenetic trees were constructed with IQ-TREE v1.6.12 [130], using -st AA -m LG+I+G4+F -bb 1000 -alrt 1000 options. Trees were visualized using iTol [131]. Bootstrap support was calculated, using an approximate likelihood ratio test (aLRT) with the Shimodaira–Hasegawa-like procedure (SH-aLRT), using 1000 bootstrap replicates.

### Data analysis (ecological statistics)

The following statistical analyses were performed in R using the Vegan [132] package: accumulation curves were calculated using the speccacum function, vOTU coverage tables were standardized using the decostand function with the Hellinger method, and Bray-Curtis dissimilarity matrices were calculated using the vegdist function. Mantel tests were performed with the mantel function, using the Pearson method, and permutational multivariate analyses of variance (PERMANOVA) were performed with the Adonis function. Venn diagrams were created with the VennDiagram package, using the draw.triple.venn function. The differential abundance analysis of vOTUs across depth levels was performed using the likelihood ratio test implemented in DESeq2 [96, 133]. Hierarchical clustering of the viral abundance patterns of the five viromes was done with the hclust function (method=complete), and heatmaps were created with the pheatmap and dendextend libraries. The world map was created with the maps library.

### Detection of putative viral auxiliary metabolic genes (AMGs)

VIBRANT [40] and DRAM-v [41] were used to identify putative AMGs in SPRUCE vOTU sequences. Briefly, these tools consider gene annotation in order to identify genes in the input contigs (in this case, our vOTUs) that have predicted functions in cellular metabolism [40, 41]. Since there is no standardized approach for AMG identification, we sought to compare results from both tools. VIBRANT was run (using standard settings) on all SPRUCE viral contigs that we had previously identified by either VirSorter or DeepVirFinder (*n*=2,802 vOTUs). Because DRAM-v requires VirSorter output, we could not use all of the DeepVirFinder-derived vOTUs. We re-ran the 4326 SPRUCE vOTUs through VirSorter, resulting in 3780 vOTUs, of which 2645 also appeared in the VIBRANT output. DRAM-v was applied (using standard settings) to these 2645 vOTUs. VIBRANT output was manually screened to determine whether predicted AMGs had viral genes upstream and downstream [15], and in many cases, they did not (see supplementary discussion). DRAM-v includes an analysis to assess the presence of viral genes upstream and downstream of the putative AMG, producing an ‘auxiliary score’ as a measure of confidence in the AMG prediction. From the DRAM-v output, only putative AMGs with auxiliary scores < 4 were retained (a low auxiliary score indicates a gene that is confidently viral), and no viral flag (F), transposon flag (T), viral-like peptidase (P), or attachment flag (A) could be present. Putative AMGs that did not have a gene ID or a gene description were also discarded. See supplemental discussion for more information.

## Supplementary Information

**Additional file 1: Supplementary figure 1**: Sampling locations for all SPRUCE samples. Sampling locations within the S1 Bog at the Marcell Experimental Forest in Northern Minnesota, USA, including the five transect samples and the samples from the SPRUCE experimental chambers. Numbers next to the brackets show how many and what kinds of metagenomes were derived from each part of the bog. **Supplementary figure 2:** A: Network of shared predicted protein content between recovered SPRUCE viruses (*n* = 4,326), and RefSeq prokaryotic viral genomes (*n* = 37). Colored nodes represent vOTUs, nodes are colored by the dataset(s) from which they were recovered, and the shared edges represent shared predicted protein content. B: Distribution of vOTUs into VCs, recovered from each of the three extraction methods and collection dates. Numbers represent number of VCs that contain vOTUs from the extraction method(s) listed. **Supplementary Figure 3:** Taxonomic classification of soil vOTUs in PIGEON. Taxonomic classifications were based on vConTACT2.0 clustering with RefSeq prokaryotic viral genomes. Percentages at the top of each graph indicate the proportion of vOTUs that were taxonomically classified, n represents the total number of vOTUs that could be taxonomically classified. **Supplementary figure 4:** Comparison of vOTU recovery from five paired viromes and total soil metagenomes from the SPRUCE transect. A: Distribution of vOTUs recovered by each of the two extraction methods, based on read mapping to the PIGEON database, including all vOTUs recovered from SPRUCE. B: Accumulation curves of distinct vOTUs recovered as sampling increases for each extraction method; 100 permutations of sample order are depicted as open circles, and averages are shown as a line. C: Similar to panel B, but only the accumulation curve of distinct vOTUs recovered from total soil metagenomes is shown, with a smaller y-axis maximum to better show the trend. **Supplementary figure 5:** Comparison of the five viromes from the transect. A: Dendrogram depicting sample similarity according to viral community composition (left) and heatmap (right) of vOTUs detected (green = detected, white = not detected) in the five SPRUCE transect viromes. B:Comparison of vOTU recovery from the SPRUCE-2 sample compared to the four other virome samples.

**Additional file 2.** All supplemental tables that are referenced in the text. Each sheet is a separate supplemental table.

**Additional file 3.** Supplemental discussion and methods

**Additional file 4.** Output of IQ-tree for the TerL phylogenetic trees, with bootstrapping values for each of the branches.

## Data Availability

The raw sequencing datasets from the SPRUCE transect have been deposited in the NCBI Sequence Read Archive (BioProject PRJNA666221) and the 5006 vOTUs assembled from SPRUCE have been deposited at DDBJ/ENA/GenBank under BioProject PRJNA706761, with accession numbers JAFMOA010000001–JAFMOA010005006. The 4326 detected vOTUs and 486 MAGs from SPRUCE and the PIGEON database (v1.0) are also available at Dryad (*https://datadryad.org/*, by DOI of the bioRxiv preprint of this paper: 10.1101/2020.12.15.422944). Sequencing data from the 82 SPRUCE experiment metagenomes were downloaded from the SPRUCE website (*https://mnspruce.ornl.gov/node/622**,* *https://mnspruce.ornl.gov/node/727*, accessed June 2019, Table S13), where they were still available at the time of manuscript submission. In addition, these 82 metagenomes are available from the JGI Genome Portal and NCBI Sequence Read Archive (SRA) with identifiers provided in Table S13. Relevant processed data and geochemical data are available as Tables S12 and S13. The code for processing viromic data and all relevant R and python scripts are available on GitHub (*https://github.com/AnneliektH/SPRUCE*).
