# Correction to: Minnesota peat viromes reveal terrestrial and aquatic niche partitioning for local and global viral populations

**DOI:** 10.1186/s40168-021-01210-x

**Published:** 2021-12-15

**Authors:** Anneliek M. ter Horst, Christian Santos-Medellín, Jackson W. Sorensen, Laura A. Zinke, Rachel M. Wilson, Eric R. Johnston, Gareth Trubl, Jennifer Pett-Ridge, Steven J. Blazewicz, Paul J. Hanson, Jeffrey P. Chanton, Christopher W. Schadt, Joel E. Kostka, Joanne B. Emerson

**Affiliations:** 1 Department of Plant Pathology, University of California, Davis, CA USA; 2 Department of Earth, Ocean, and Atmospheric Science, Florida State University, Tallahassee, FL USA; 3 Biosciences Division, Oak Ridge National Laboratory, Oak Ridge, TN USA; 4 Physical and Life Sciences Directorate, Lawrence Livermore National Laboratory, Livermore, CA USA; 5 Environmental Sciences Division, Oak Ridge National Laboratory, Oak Ridge, TN USA; 6 Schools of Biology and Earth & Atmospheric Sciences, Georgia Institute of Technology, Atlanta, GA USA; 7 Center for Microbial Dynamics and Infection, Georgia Institute of Technology, Atlanta, GA 30332 USA; 8 Genome Center, University of California, Davis, CA USA

**Correction to: Microbiome 9, 233 (2021)**

**https://doi.org/10.1186/s40168-021-01156-0**

Following the publication of the original article [1], the co-author reported that his name should be “Gareth Trubl” (no middle initial G) instead of “Gareth G. Trubl”.

The original article has been updated to correct this.
