# Correction to: Minnesota peat viromes reveal terrestrial and aquatic niche partitioning for local and global viral populations

**DOI:** 10.1186/s40168-022-01229-8

**Published:** 2022-01-25

**Authors:** Anneliek M. ter Horst, Christian Santos-Medellín, Jackson W. Sorensen, Laura A. Zinke, Rachel M. Wilson, Eric R. Johnston, Gareth Trubl, Jennifer Pett-Ridge, Steven J. Blazewicz, Paul J. Hanson, Jeffrey P. Chanton, Christopher W. Schadt, Joel E. Kostka, Joanne B. Emerson

**Affiliations:** 1 Department of Plant Pathology, University of California Davis, Davis, CA USA; 2 Department of Earth, Ocean, and Atmospheric Science, Florida State University, Tallahassee, FL USA; 3 Biosciences Division, Oak Ridge National Laboratory, Oak Ridge, TN USA; 4 Physical and Life Sciences Directorate, Lawrence Livermore National Laboratory, Livermore, CA USA; 5 Environmental Sciences Division, Oak Ridge National Laboratory, Oak Ridge, TN USA; 6 Schools of Biology and Earth & Atmospheric Sciences, Georgia Institute of Technology, Atlanta, GA USA; 7 Center for Microbial Dynamics and Infection, Georgia Institute of Technology, Atlanta, GA 30332 USA; 8 Genome Center, University of California Davis, Davis, CA USA

**Correction to: Microbiome 9, 233 (2021)**

**https://doi.org/10.1186/s40168-021-01156-0**

Following the publication of the original article [1], the co-author reported that Funding section was not included. Below is the Funding section.

Funding for this work was provided by the UC Davis College of Agricultural and Environmental Sciences and Department of Plant Pathology as new lab start-up to JBE (for research expenses and the majority of support for AMH). Additional support for AMH was provided by an award from the U.S. Department of Energy (DOE), Office of Science, Office of Biological and Environmental Research (BER), Genomic Science Program, Number DE-SC0021198 (grant to JBE). Support for CSM was provided by an award from the DOE BER, Genomic Science Program, Number DE-SC0020163 (grant to JBE). Support for PJH and CWS was provided by the U.S. Department of Energy, Office of Science, Office of Biological and Environmental Research. ORNL is managed by UT-Battelle, LLC, for the DOE under contract DE-AC05–1008 00OR22725. Contributions by RMW, JPC, and JEK were supported by the Office of Biological and Environmental Research, Terrestrial Ecosystem Science Program, under United States DOE contracts DE-SC0007144 and DE-SC0012088 (grants to JEK). Data collection for the Alaskan samples was supported by the USGS Mendenhall Postdoctoral Fellowship program and for the Puerto Rico samples by DOE BER Early Career Research Program grant SCW1478 (to JPR). Analyses and data collection conducted by Lawrence Livermore National Laboratory (LLNL) were conducted under the auspices of DOE Contract DE-AC52-07NA27344 and supported by the DOE BER Genomic Science Soil Microbiome SFA SCW1632 and LLNL LDRD 18-ERD-041 (to SJB). Sequencing for the Puerto Rico samples was supported by JGI Community Sequencing Award #2017 (JGI project ID #502924) and several NERSC allocations (to JPR).

The original article has been updated.
