# Supplementary material for: Minnesota peat viromes reveal terrestrial and aquatic niche partitioning for local and global viral populations
Source: Microbiome. 2021 Nov 26;9:233. doi: 10.1186/s40168-021-01156-0 (PMC8626947; doi:10.1186/s40168-021-01156-0)
Supplement: Supplementary file 2 — Additional file 1: Supplementary figure 1: Sampling locations for all SPRUCE samples. Sampling locations within the S1 Bog at the Marcell Experimental Forest in Northern Minnesota, USA, including the five transect samples and the samples from the SPRUCE experimental chambers. Numbers next to the brackets show how many and what kinds of metagenomes were derived from each part of the bog. Supplementary figure 2: A: Network of shared predicted protein content between recovered SPRUCE viruses (n = 4,326), and RefSeq prokaryotic viral genomes (n = 37). Colored nodes represent vOTUs, nodes are colored by the dataset(s) from which they were recovered, and the shared edges represent shared predicted protein content. B: Distribution of vOTUs into VCs, recovered from each of the three extraction methods and collection dates. Numbers represent number of VCs that contain vOTUs from the extraction method(s) listed. Supplementary Figure 3: Taxonomic classification of soil vOTUs in PIGEON. Taxonomic classifications were based on vConTACT2.0 clustering with RefSeq prokaryotic viral genomes. Percentages at the top of each graph indicate the proportion of vOTUs that were taxonomically classified, n represents the total number of vOTUs that could be taxonomically classified. Supplementary figure 4: Comparison of vOTU recovery from five paired viromes and total soil metagenomes from the SPRUCE transect. A: Distribution of vOTUs recovered by each of the two extraction methods, based on read mapping to the PIGEON database, including all vOTUs recovered from SPRUCE. B: Accumulation curves of distinct vOTUs recovered as sampling increases for each extraction method; 100 permutations of sample order are depicted as open circles, and averages are shown as a line. C: Similar to panel B, but only the accumulation curve of distinct vOTUs recovered from total soil metagenomes is shown, with a smaller y-axis maximum to better show the trend. Supplementary figure 5: Comparison of the five viromes [file 40168_2021_1156_MOESM2_ESM.pdf]

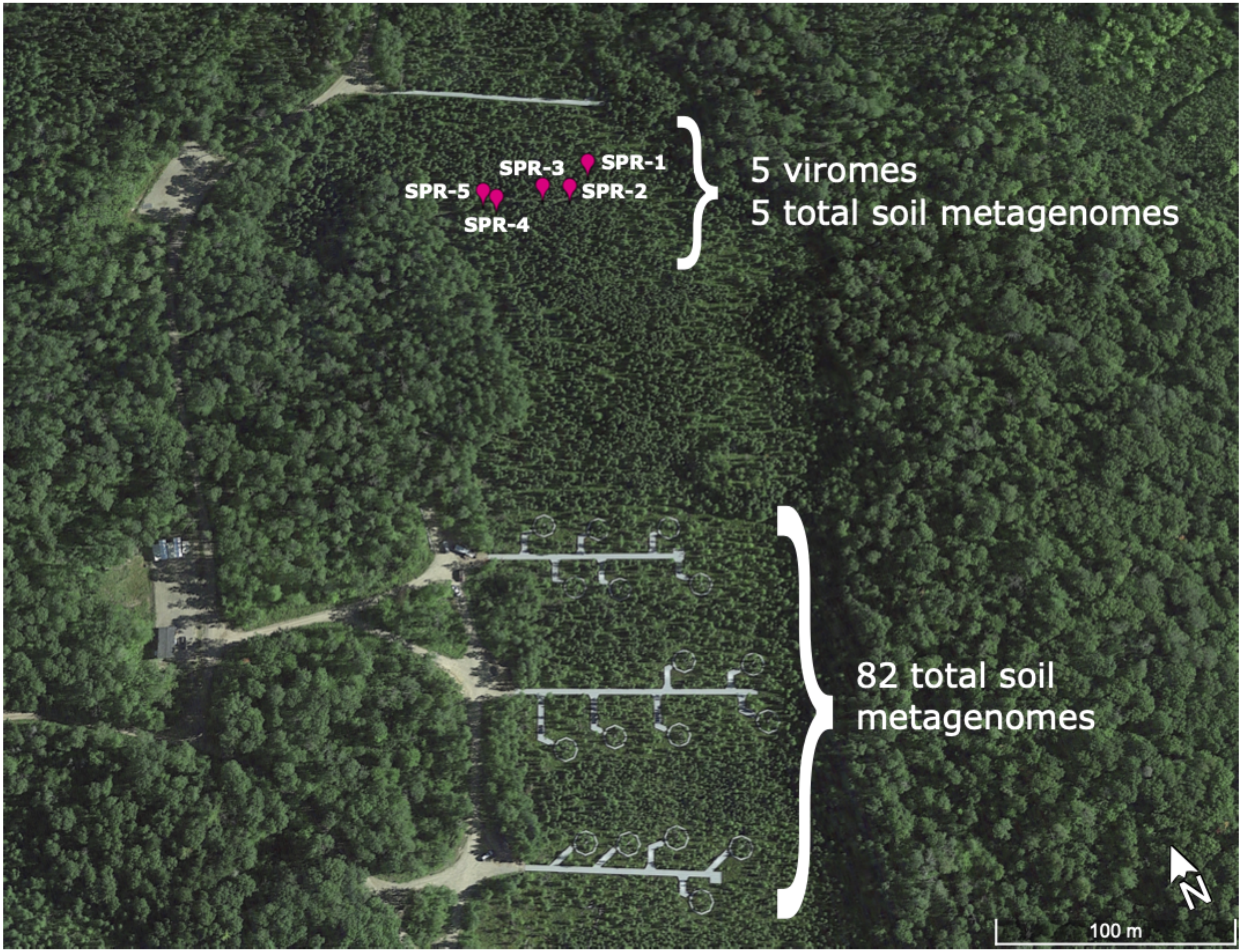

Supplementary figure 1: **Sampling locations for all SPRUCE samples**. Sampling locations within the S1 Bog at the Marcell Experimental Forest in Northern Minnesota, USA, including the five transect samples and the samples from the SPRUCE experimental chambers. Numbers next to the brackets show how many and what kinds of metagenomes were derived from each part of the bog.

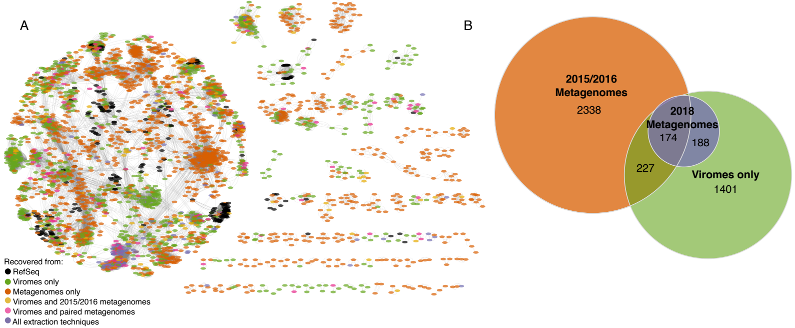

Supplementary figure 2: A: Network of shared predicted protein content between recovered SPRUCE viruses (n = 4,326), and RefSeq prokaryotic viral genomes (n = 37). Colored nodes represent vOTUs, nodes are colored by the dataset(s) from which they were recovered, and the shared edges represent shared predicted protein content. **B**: Distribution of vOTUs into VCs, recovered from each of the three extraction methods and collection dates. Numbers represent number of VCs that contain vOTUs from the extraction method(s) listed.

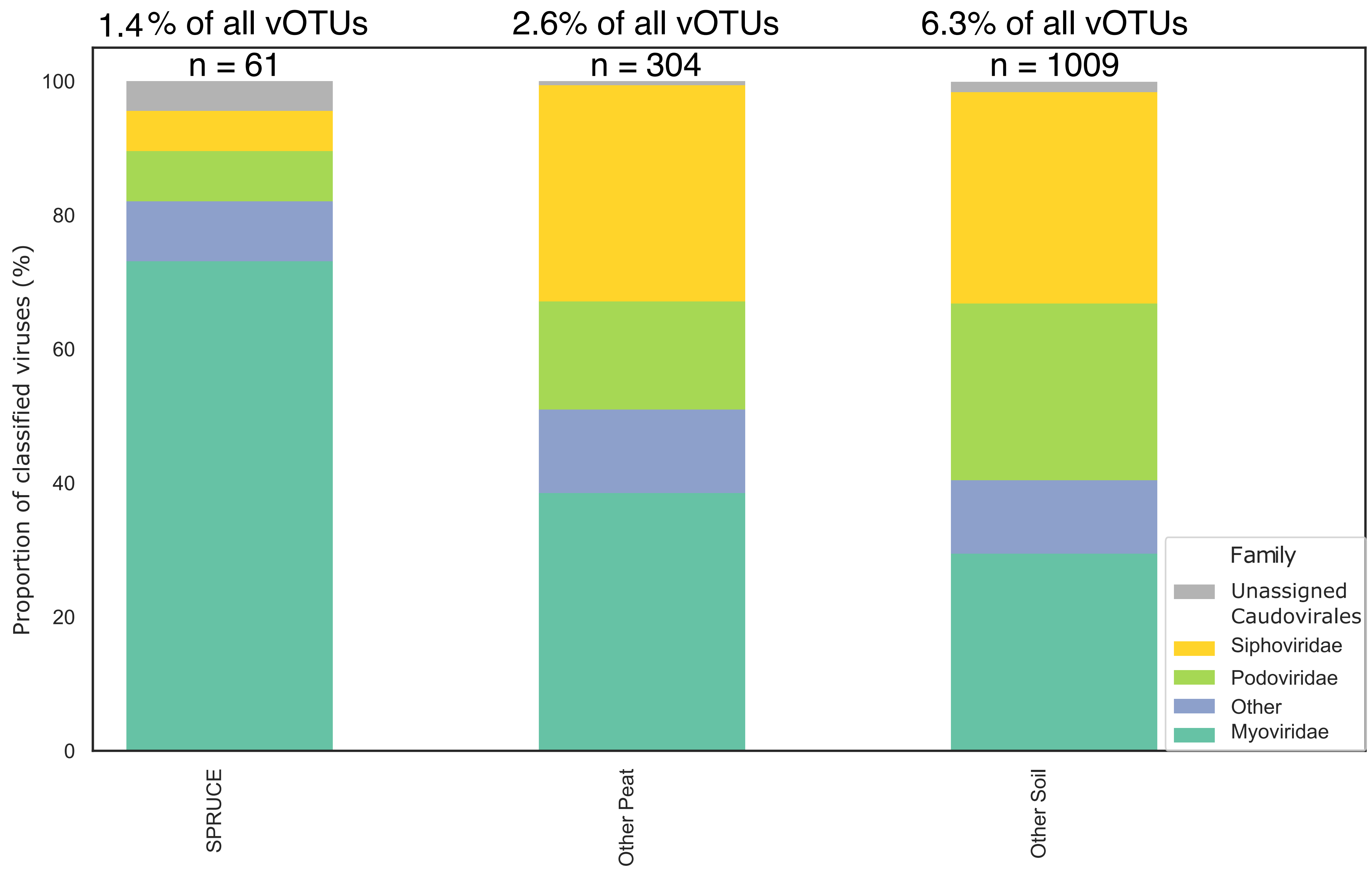

Supplementary Figure 3: Taxonomic classification of soil vOTUs in PIGEON. Taxonomic classifications were based on vConTACT2.0 clustering with RefSeq prokaryotic viral genomes. Percentages at the top of each graph indicate the proportion of vOTUs that were taxonomically classified, n represents the total number of vOTUs that could be taxonomically classified.

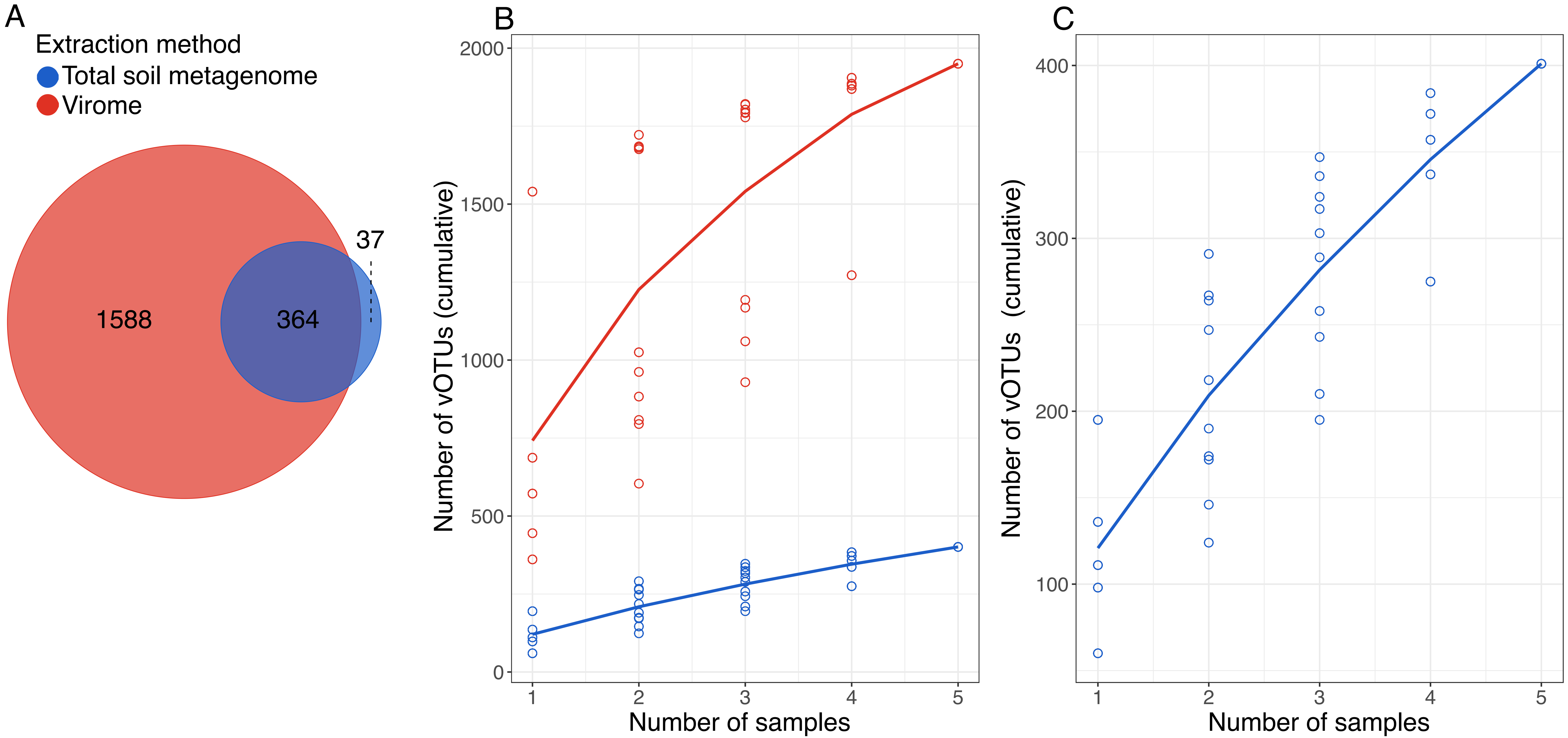

Supplementary figure 4: **Comparison of vOTU recovery from five paired viromes and total soil metagenomes from the SPRUCE transect. A**: Distribution of vOTUs recovered by each of the two extraction methods, based on read mapping to the PIGEON database, including all vOTUs recovered from SPRUCE. **B**: Accumulation curves of distinct vOTUs recovered as sampling increases for each extraction method; 100 permutations of sample order are depicted as open circles, and averages are shown as a line. **C**: Similar to panel B, but only the accumulation curve of distinct vOTUs recovered from total soil metagenomes is shown, with a smaller y-axis maximum to better show the trend.

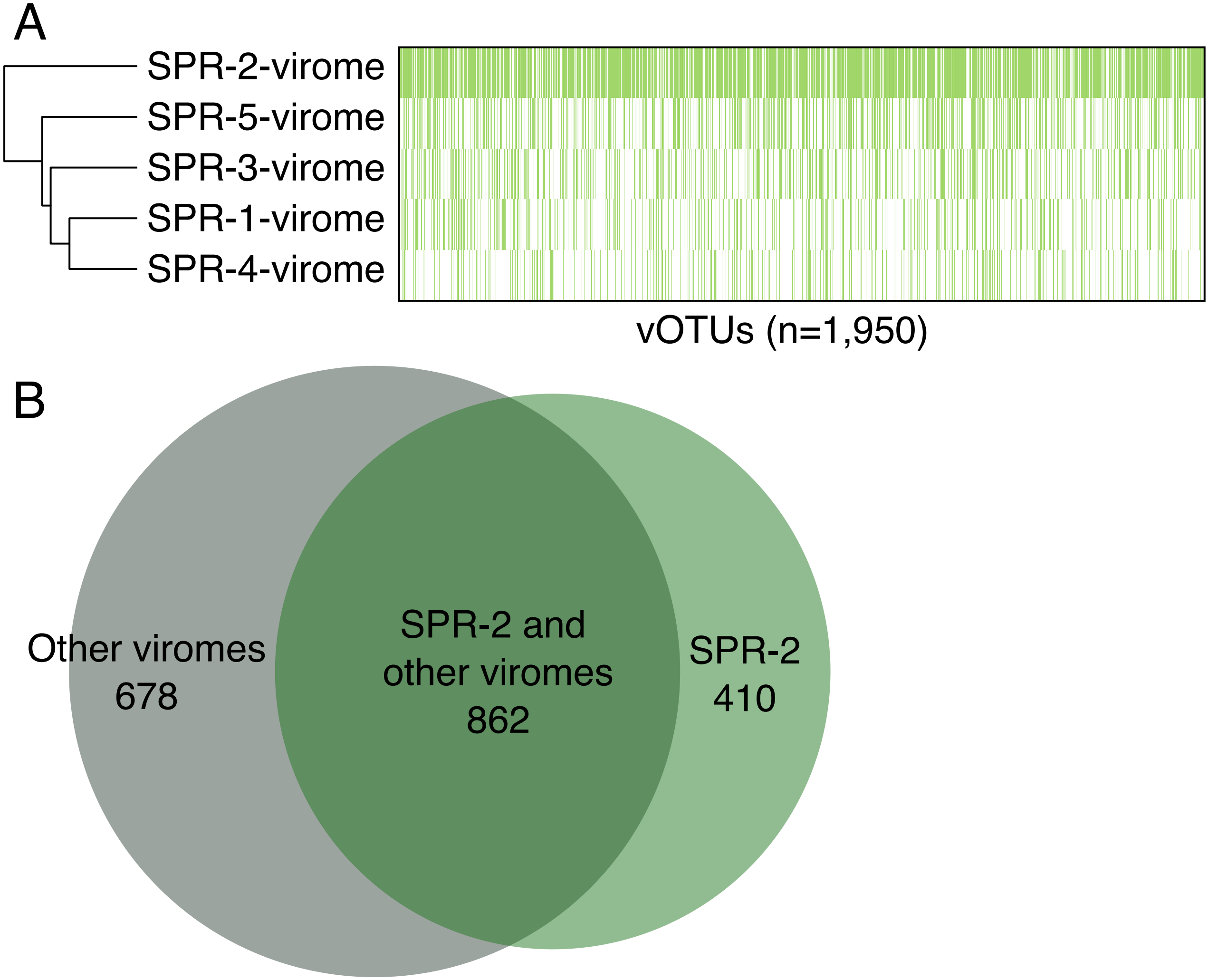

Supplementary figure 5: Comparison of the five viromes from the transect. A: Dendrogram depicting sample similarity according to viral community composition (left) and heatmap (right) of vOTUs detected (green = detected, white = not detected) in the five SPRUCE transect viromes.
B: Comparison of vOTU recovery from the SPRUCE-2 sample compared to the four other virome samples.
